# Vascular leiomyosarcoma originating from the right ovarian vein: a case report and literature review

**DOI:** 10.1186/s40792-019-0679-5

**Published:** 2019-07-24

**Authors:** Takuro Hirano, Hiroshi Okumura, Satoru Maeda, Mario Shimada, Akira Imakiire, Kanro Makisumi, Michiyo Higashi, Shoji Natsugoe

**Affiliations:** 1 Department of Surgery, Southern Region Hospital, Midorimachi 220, Makurazaki, Kagoshima 898-0011 Japan; 2 Department of Pathology, Kagoshima University Hospital, Sakuragaoka 8-35-1, Kagoshima, 890-8520 Japan; 3 Department of Digestive Surgery, Breast and Thyroid Surgery, Kagoshima University Graduate School of Medical and Dental Sciences, Sakuragaoka 8-35-1, Kagoshima, 890-8520 Japan

**Keywords:** Leiomyosarcoma, Ovarian vein, Vascular leiomyosarcoma, Retroperitoneal sarcoma

## Abstract

**Background:**

Primary leiomyosarcoma (LMS) of vascular origin is a rare lesion, and patients with LMS of vascular origin have poorer prognoses than patients with LMS of other origins. The inferior vena cava is the most commonly affected vessel and accounts for 60% of all vascular cases. However, LMS originating from the ovarian vein is extremely rare, and we are only aware of 15 reported cases. Therefore, we report our experience with a case of LMS originating from the right ovarian vein and review the related literature.

**Case presentation:**

A 71-year-old Japanese woman with no symptoms was admitted to our hospital because of abnormal findings in a routine abdominal ultrasonography check-up. Contrast-enhanced computed tomography of the abdomen revealed a well-defined, lobulated solid mass with a diameter of 5.5 cm in the right retroperitoneal space. The mass exhibited relatively low uptake during ^18^F-fluorodeoxyglucose positron emission tomography. Based on these findings, the differential diagnosis included a retroperitoneal tumor, such as a desmoid tumor, leiomyoma, LMS, and malignant mesothelioma. Operative findings confirmed that the mass had originated from the right ovarian vessels, and en bloc excision was performed for the mass and the right ovarian vessels. The final pathological diagnosis was LMS originating from the right ovarian vein, and the surgical resection margins were free from tumor cells. After histological findings confirmed the LMS diagnosis, the patient underwent adjuvant radiation therapy and has not exhibited signs of local recurrence or metastasis in the 6 months after surgery.

**Conclusions:**

We encountered a 71-year-old woman with LMS originating from her right ovarian vein. The prognosis of vascular LMS is generally poor. Therefore, careful follow-up will be required for our patient.

## Background

Leiomyosarcoma (LMS) is a mesenchymal tumor of smooth muscle origin. However, primary LMS of vascular origin is a rare lesion that represents < 1/100,000 of malignant tumors. Patients with LMS of vascular origin have poorer prognoses than patients with LMS of other origins [1]. The inferior vena cava is the most commonly affected vessel, accounting for 60% of all vascular cases [2], followed by the large central veins and the long saphenous veins [2, 3]. The ovarian veins are an extremely rare site of origin of LMS, with only 15 reported cases [2, 4–17]. Therefore, we report our experience with a case of primary LMS originating from the right ovarian vein and review the related literature.

## Case presentation

**Fig. 1:**
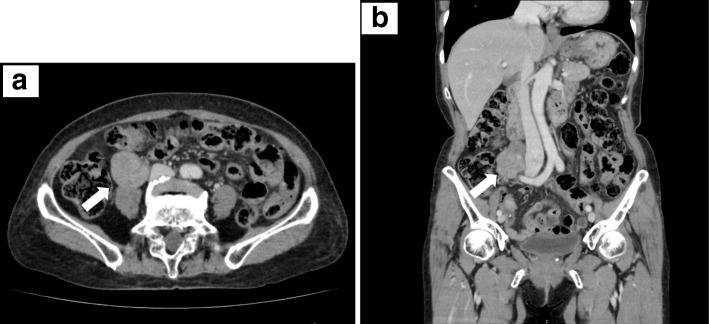
Contrast-enhanced computed tomography of the leiomyosarcoma. Axial (**a**) and coronal (**b**) contrast-enhanced computed tomography of the patient’s abdomen revealing a well-defined, lobulated solid mass (white arrows) with a diameter of 5.5 cm in the right retroperitoneal space

**Fig. 2:**
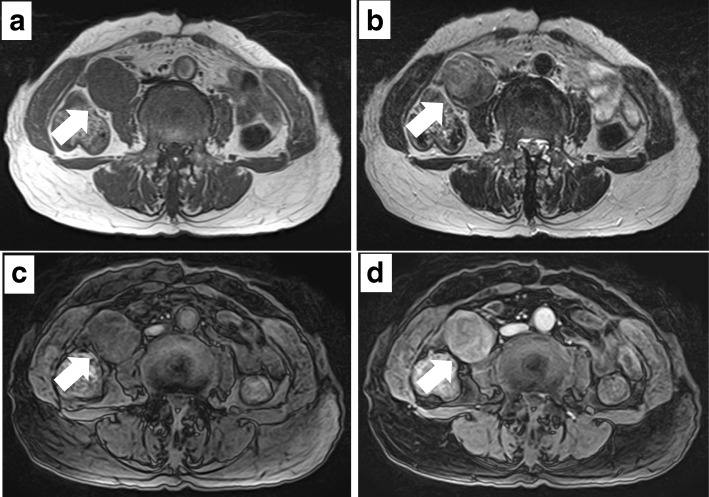
Magnetic resonance imaging of the leiomyosarcoma. **a** Axial T1-weighted magnetic resonance imaging (MRI) of the abdomen revealing a tumor (white arrows) with diffuse low intensity. **b** Axial T2-weighted MRI revealing a tumor with uniform low intensity. Early-phase (**c)** and late-phase (**d**) axial gadolinium-enhanced MRI revealing heterogeneous and incremental enhancement of the tumor

**Fig. 3:**
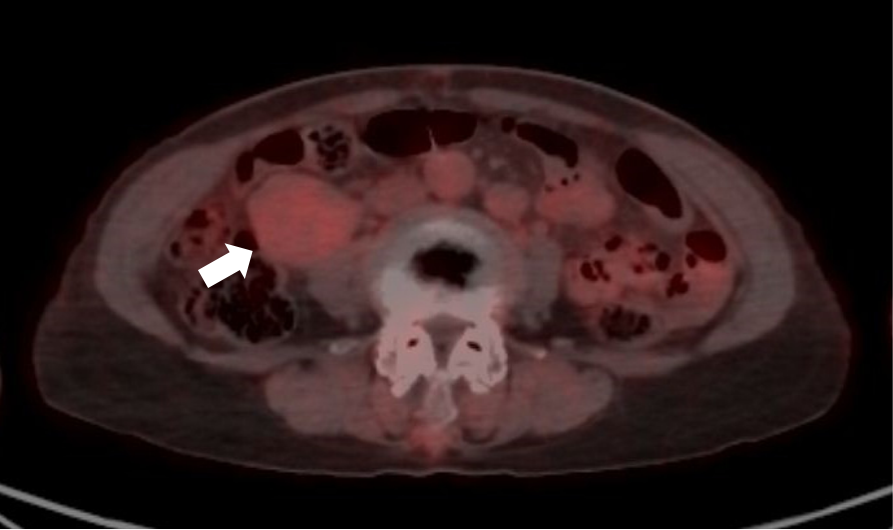
^18^F-fluorodeoxyglucose positron emission tomography (FDG-PET) of the leiomyosarcoma. Axial fusion images of FDG-PET revealing that the tumor (white arrows) had relatively low uptake

A 71-year-old previously healthy Japanese woman with no symptoms was admitted to our hospital because of abnormal findings in a routine abdominal ultrasonography check-up, which supported a suspicion of main pancreatic duct dilatation and gallbladder polyps. Physical examination revealed a palpable mass with good mobility in the right lower abdomen. The laboratory findings were all within the normal ranges. Contrast-enhanced computed tomography (CT) of the abdomen revealed no abnormal findings in the pancreas and gallbladder but revealed a well-defined, lobulated solid mass with a diameter of 5.5 cm in the right retroperitoneal space (Fig. 1a, b). Magnetic resonance imaging (MRI) of the abdomen was performed: on T1-weighted imaging, the mass showed diffuse low intensity (Fig. 2a), and on T2-weighted imaging, the mass showed uniform low intensity (Fig. 2b). On gadolinium enhancement, the mass showed heterogeneous and incremental enhancement (Fig. 2c, d). On ^18^F-fluorodeoxyglucose positron emission tomography (FDG-PET), the mass had relatively low uptake, with a maximum standardized uptake value (SUVmax) of 2.4 (Fig. 3). Based on these findings, the differential diagnosis included a retroperitoneal tumor, such as a desmoid tumor, leiomyoma, LMS, and malignant mesothelioma.

**Fig. 4:**
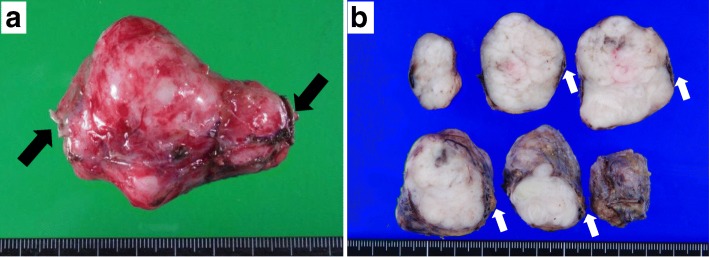
Gross findings of the leiomyosarcoma. **a**, **b** The specimen consisting of a white solid tumor that measured 5.5 cm × 4.5 cm. **b** The ovarian vein (white arrow) can be seen passing through the dorsal side of the tumor

**Fig. 5:**
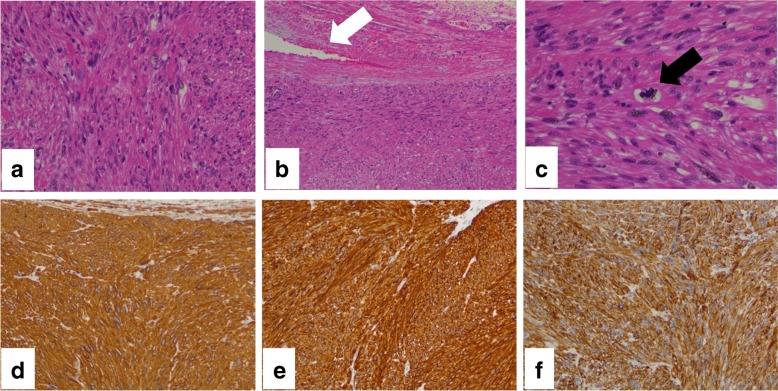
Histological findings of the leiomyosarcoma. **a** Hematoxylin and eosin staining (HE, × 200) showing atypical spindle cells with interlacing fascicles and **b** tumor cells connected to the right ovarian vein (white arrow; HE, × 100). **c** Mitosis in the tumor cell (black arrow; HE, × 400). Immunohistochemical staining (× 100) showed positive results for **d** α-SMA, **e** desmin, and **f** caldesmon

Exploratory laparotomy was performed through a median incision, and the operative findings confirmed that the mass was attached and connected to the right ovarian vessels. The mass was slightly adhered to the ureter, but there were no signs of invasion to the other adjacent organs. Because the mass appeared to originate from the right ovarian vessels, en bloc excision of the mass and right ovarian vessels was performed. The surgical specimen consisted of a white solid tumor (5.5 cm × 4.5 cm) (Fig. 4a, b); the ovarian vessels passed through the dorsal side of the tumor (Fig. 4b). Microscopically, atypical spindle cells revealed interlacing fascicles without necrosis (Fig. 5a) and that the tumor cells connected with the right ovarian vein (Fig. 5b) with 50 mitoses per 50 high-power fields (HPF) (Fig. 5c). On immunohistochemical staining, the tumor cells were found to be diffusely positive for α-smooth muscle actin (α-SMA) (Fig. 5d), desmin (Fig. 5e), and caldesmon (Fig. 5f) and negative for CD34, S100, c-kit, MDM2, and cyclin-dependent kinase 4 (CDK4). These findings supported a diagnosis of LMS. The surgical resection margins were free from tumor cells. The patient’s postoperative course and recovery were uneventful. Based on the histological diagnosis of LMS, the patient underwent adjuvant radiation therapy (total dose, 50.4 Gy in 25 fractions) and has not exhibited signs of local recurrence or metastasis in the 6 months after surgery.

## Discussion

LMS is the second most common retroperitoneal sarcoma among adults [18]. Retroperitoneal LMS originates from smooth muscles within the retroperitoneum, such as the wall of the retroperitoneal veins or embryonic remnants [19]. The vena cava is the most commonly affected vessel in vascular LMS cases, and these cases have been well described. The ovarian vein is the eighth most commonly affected vessel in cases of vascular LMS that do not originate from the vena cava [20]. Therefore, LMS originating from the ovarian vein is extremely rare, and we are only aware of 15 reported cases [2, 4–17]. Table 1 summarizes the 15 reported cases and our case. Median patient age at diagnosis was 55 years (range, 37–78 years) and median maximum tumor size was 7.5 cm (range, 3–28 cm). Twelve of the 15 reported cases (80%) involved nonspecific symptoms, including abdominal pain (6 cases), abdominal mass (4 cases), abdominal distension (1 case), and abdominal discomfort (1 case). Two patients complained of symptoms related to the tumor’s invasion of the ureter (repeated pyelonephritis and back pain). Our patient is the only one to have no symptoms at diagnosis.

**Table 1:** Summary of leiomyosarcoma originated from the ovarian vein

Case		16
Age (years)* (*n* = 16)		55 (37–71)
Tumor size (cm)* (*n* = 15)		7.5 (3–28)
Symptom (%) (*n* = 15)		
	Abdominal pain	6 (40)
	Abdominal mass	4 (26.7)
	Pyelonephritis	1 (6.7)
	Abdominal distension	1 (6.7)
	Back pain	1 (6.7)
	Abdominal discomfort	1 (6.7)
	No symptom	1 (6.7)
Side (%) (*n* = 15)	Left:right	6 (40):9 (60)
Growth pattern (%) (*n* = 9)	extra/intra/mixed	4 (44.4)/0 (0)/5 (55.6)
Modality (%) (*n* = 13)	CT/MRI/PET	13 (100)/5 (38.5)/3 (23.1)
Preoperative diagnosis (%) (*n* = 13)	Yes/no	4 (30.8)/9 (69.2)
Surgery (%) (*n* = 16)		
	Simple tumor excision	2 (12.5)
	En block	5 (31.2)
	En block with adjacent organs	9 (56.3)
Adjuvant therapy (%) (*n* = 14)	Chemotherapy/radiotherapy/no	3 (21.4)/4 (28.6)/7 (50.0)
Recurrence (%) (*n* = 12)	Yes/no	6 (50.0)/6 (50.0)
Recurrence pattern (%) (*n* = 12)	Local recurrece/distant metastasis	1 (8.3)/5 (41.7)
Metastatic site (%) (*n* = 11)	Lung/liver/others	4 (36.4)/3 (27.2)/4 (36.4)
DFS (months)* (*n* = 11)		12 (2–44)
Follow-up (months)* (*n* = 12)		12 (2–44)

*Median

*CT* Computed tomography, *MRI* magnetic resonance imaging, *FDG-PET* 18F-fluorodeoxyglucose positron emission tomography

Tumors originating from the left ovarian vein and from the right ovarian vein have been reported in 6 (40%) and 9 (60%) cases, respectively.

Three major growth patterns have been reported for retroperitoneal LMS: completely extravascular (extraluminal, 62% of cases), completely intravascular (intraluminal, 5% of cases), and a combination of extraluminal and intraluminal patterns (mixed, 33% of cases) [21]. Cases of LMS with an extraluminal pattern are usually diagnosed late in their course, while LMS cases with intraluminal and mixed patterns are more likely to show early symptoms that depend on the affected vein [21]. Among 9 reports on LMS originating from the ovarian veins, extraluminal patterns were observed in 4 cases (44.4%) and mixed patterns in 5 cases (55.6%), with no cases involving intraluminal patterns. In the present case, the growth pattern was considered to be extraluminal because the ovarian vein passed through the dorsal side of the tumor and because no tumor cells had invaded the ovarian vein lumen.

Based on these presentations, contrast-enhanced CT is especially useful for establishing the preoperative diagnosis and for determining the tumor extent and boundaries before starting surgery [15]. Previous reports have indicated that CT typically reveals a large mass with heterogeneous contrast in the longitudinal orientation, which replaces the normal ovarian vein as well as cystic and necrotic components and vascular hypertrophy [8, 15, 22]. Additional information regarding the lesion can be obtained using MRI [9, 10]. Previous reports have also indicated that increased uptake during FDG-PET provided high accuracy for diagnosing LMS [23]. FDG-PET can be used to diagnose a retroperitoneal mass as an LMS originating from an ovarian vein [10]. Among the 13 cases of LMS originating from the ovarian vein, CT was performed in 13 (100%), MRI was performed in 5 (38.5%), and PET was performed in 3 (23.1%); preoperative diagnosis was made in 4 cases (30.8%). In our case, contrast-enhanced CT revealed a solid mass with no necrosis, which is less common for extraluminal LMS but typical for smaller retroperitoneal tumors [21]. Furthermore, the mass exhibited relatively low uptake during FDG-PET, which made it difficult to establish a preoperative diagnosis of LMS. In our case, the tumor was detected early, was small in size, and had a low mitotic rate. Therefore, the findings (no necrosis and low FDG accumulation) were not typical.

In retroperitoneal sarcoma cases, 60% of resected adjacent organs were found to be microscopically invaded by the tumor [10]. En bloc resection with histopathologically free margins is essentially the only procedure that can result in a good prognosis [15, 24]. However, even after complete tumor resection, patients with retroperitoneal sarcoma have poor prognoses, based on a > 50% recurrence rate and a 5-year survival rate of 52–65% [18, 25, 26]. Thus, in cases of large soft tissue sarcomas with high-grade histological findings, radiation therapy and chemotherapy are considered before or after surgery to lower the risk of local recurrence and distant metastases [27, 28]. Therefore, we performed adjuvant radiation therapy to lower the risk of local recurrence. If LMS originating from the ovarian vein had been diagnosed preoperatively in our case, comprehensive treatment with a combination of en block excision of the tumor and ovarian vessels and adjuvant therapy would have been recommended.

Metastasis is identified at diagnosis in 12% of cases of vascular LMS that did not originate from the vena cava [20], although none of the reported cases of LMS originating from the ovarian vein involved metastasis at the initial diagnosis. All of these patients underwent radical resection that did or did not involve the adjacent organs; 50% of patients (7/14) received adjuvant radiation therapy (28.6%, 4/14) or chemotherapy (21.4%, 3/14). Tumor recurrence after radical resection was observed in 50% of cases (6/12). Distant metastasis (41.7%, 5/12) was more frequent than local recurrence (8.3%, 1/12), and the popular metastatic sites were the lung (36.4%, 4/11) and liver (27.2%, 3/11). These findings are consistent with the previously reported characteristics of retroperitoneal LMS [18, 29]. Median interval to tumor recurrence was 12 months (range, 2–17 months), and the shortest and longest recurrence-free periods were 2 months and 17 months, respectively; therefore, surveillance every 3 months for at least 2 years is recommended. Table 2 shows the correlation between tumor recurrence and clinicopathological factors based on the literature. Interestingly, all patients with > 20 mitoses/10 HPF eventually developed recurrence after radical resection, whereas no metastasis was detected in cases with < 20 mitoses/10 HPF. Thus, a low mitotic rate may predict a favorable prognosis; however, for cases without recurrence, the median observation period was only 13.5 months, which is too short for an accurate evaluation of the patients’ prognoses. In addition, younger age and tumor resection involving the adjacent organs may be potential risk factors for recurrence; however, analysis of recurrence showed no significant risk factors, which may be due to the small cohort size.

**Table 2:** Correlation between tumor recurrence and clinicopathological factors in patients with leiomyosarcoma originating from ovarian vein

	*n* (%)	Tumor recurrence		*P* value
		Yes	No	
Patient, *n* (%)	12	6 (50.0)	6 (50.0)	
Age (range)	12	51 (37–67)	59.5 (56–78)	0.09
Side, *n* (%)	11			
Left	6 (54.5)	2 (40.0)	4 (66.7)	0.57
Right	5 (45.5)	3 (60.0)	2 (33.3)	
Tumor size (range)	11	16.5 (3–28)	7.3 (5–21)	0.32
Growth pattern, *n* (%)	9			
Extra	4 (44.4)	1 (20.0)	3 (75.0)	0.21
Mixed	5 (55.6)	4 (80.0)	1 (25.0)	
Surgery, *n* (%)	12			
Tumor resection (simple excision + en block)	5 (41.7)	1 (16.7)	5 (83.3)	0.08
Tumor resection with adjacent organ	7 (58.3)	5 (83.3)	1 (16.7)	
Adjuvant therapy, *n* (%)	11			
Yes	5 (45.5)	2 (40.0)	3 (50.0)	1
No	6 (54.5)	3 (60.0)	3 (50.0)	
Mitotic count, *n* (%)	7			
< 20/HPF	4 (57.1)	0 (0.0)	4 (100)	0.03
≥ 20/HPF	3 (42.9)	3 (100)	0 (0.0)	

Statistical analyses of two groups were performed using *χ*^2^-test

In the present case, careful follow-up will be required, despite the low mitotic rate of 10 mitoses/10 HPF, which would predict a good outcome based on the previous sentence.

## Conclusion

We encountered a 71-year-old woman with rare vascular LMS originating from the right ovarian vein. Although the prognosis of LMS originating from the ovarian vein is obscured by its rarity and short-term observation data, the prognosis of vascular LMS is generally poor. Therefore, careful follow-up will be required for our patient.

## Data Availability

All related data are included within the article.

## Abbreviations

CDK4: Cyclin-dependent kinase 4
CT: Computed tomography
FDG-PET: ^18^F-fluorodeoxyglucose positron emission tomography
HPF: High-power field
LMS: Leiomyosarcoma
MRI: Magnetic resonance imaging
α-SMA: α-smooth muscle actin
